# Sphingolipid and Trimethylamine-N-Oxide (TMAO) Levels in Women with Obesity after Combined Physical Training

**DOI:** 10.3390/metabo14080398

**Published:** 2024-07-23

**Authors:** Camila Fernanda Cunha Brandao, Michel Krempf, Flávia Giolo de Carvalho, Audrey Aguesse, Márcia Varella Morandi Junqueira-Franco, Gabriela Batitucci, Ellen Cristini de Freitas, Natalia Yumi Noronha, Guilherme da Silva Rodrigues, Gizela Pedroso Junqueira, Diego Alcantara Borba, Stéphanie Billon-Crossouard, Mikael Croyal, Julio Sergio Marchini

**Affiliations:** 1 Ribeirão Preto Medical School, University of São Paulo, Av. Bandeirantes 3900, Ribeirão Preto 14000-000, São Paulo, Brazil; mvmjf@hotmail.com (M.V.M.J.-F.); nataliayumi@usp.br (N.Y.N.); guirodrigues@usp.br (G.d.S.R.); gizelajunqueira@usp.br (G.P.J.); 2 Department of Physical Education, State University of Minas Gerais, Divinópolis 35500-000, Minas Gerais, Brazil; diego.alcantara@uemg.br; 3 NUN, INRA, The Research Unit of the Thorax Institute, CHU Nantes, UMR 1280, PhAN, IMAD, CRNH-O, F-44000 Nantes, France; michel.krempf@univ-nantes.fr (M.K.); audrey.aguesse@gmail.com (A.A.); stephanie.crossouard@univ-nantes.fr (S.B.-C.); mikael.croyal@univ-nantes.fr (M.C.); 4 School of Physical Education and Sport of Ribeirão Preto, University of São Paulo, Av. Bandeirantes 3900, Ribeirão Preto 14000-000, São Paulo, Brazil; flaviagiolo@gmail.com (F.G.d.C.); ellenfreitas@usp.br (E.C.d.F.); 5 Department of Food and Nutrition, School of Pharmaceutical Sciences of Araraquara, State University of São Paulo, Rod. Araraquara–Jau Km 1, Araraquara 14800-000, São Paulo, Brazil; gabibatitucci@gmail.com

**Keywords:** physical fitness, body composition, lipids, cardiovascular risk, weight loss

## Abstract

Obesity causes metabolic changes, such as the development of cardiovascular diseases. Moreover, physical exercise promotes protection against these diseases. Thus, the objective of the present study was to evaluate whether combined physical training can improve the metabolic system of women with obesity, reducing plasma concentrations of trimethylamine N-oxide (TMAO) and sphingolipids, regardless of weight loss. Fourteen obese women (BMI 30–40 kg/m^2^), aged 20–40 years, sedentary, were submitted to 8 weeks of combined physical training (strength and aerobic exercises). The training was performed three times/week, 55 min/session, at 75–90% maximum heart rate. All participants were evaluated pre- and post-exercise intervention, and their body composition, plasma TMAO, creatinine, lipid profile, and sphingolipid concentrations were recorded. Maximum oxygen consumption (VO2max), Speed lactate threshold 1 (SpeedLT1), and Speed lactate threshold 2 (SpeedLT2) evaluated physical performance. Results: After combined exercise, it did not change body composition, but TMAO, total cholesterol, and sphingolipid concentrations significantly decreased (*p* < 0.05). There was an increase in physical performance by improving VO2max, SpeedLT1, and SpeedLT2 (*p* < 0.05). The combined physical exercise could induce cardiovascular risk protection by decreasing TMAO in obese women, parallel to physical performance improvement, independent of weight loss.

## 1. Introduction

Obesity is considered a global epidemic, with an estimated 2.1 billion people worldwide being obese or overweight, representing almost 30% of the world’s population [1]. Traditionally, obesity is seen as a high energy intake and a sedentary lifestyle, resulting in a positive energy balance that will be stored as energy in adipose tissue [2,3]. However, obesity is much more complex, as several internal and external factors contribute to this growing challenge [4,5].

Obesity is also a significant risk factor for the development of cardiovascular diseases [6,7]. Human studies have shown positive associations between trimethylamine N-oxide (TMAO), body mass index (BMI), and fat mass (FM) [8,9]. TMAO has been identified as a biomarker of cardiovascular morbidity (CVD) risk factors [10]. The concentration of TMAO originates through trimethylamine and intestinal bacterial flora from dietary compounds of carnitine, betaine, and choline [11]. A landmark study [12,13] reported that higher plasma concentrations of TMAO were associated with a 50% increase in the adverse event of coronary morbidity. Furthermore, many recent meta-analysis data have confirmed that circulating TMAO levels predict increases in cardiovascular disease and mortality risks. Each 10 µM increase in TMAO level is associated with an approximate 7.6% increase in the relative risk of all-cause mortality [14,15]. They have also been linked to increased cholesterol deposition in macrophages and the development of atherosclerosis [10]. Furthermore, elevated circulating TMAO produces pro-inflammatory cytokines, contributing to the induction of obesity [6,15].

In this same sense, inflammation caused by obesity is systematically amplified throughout the body [16], which can activate the synthesis of lipids such as sphingolipids, mainly due to the increase in free fatty acids in the blood circulation, which assists the synthesis of ceramides through the de novo pathway. High levels of ceramides have been associated with obesity and other metabolic diseases [17]. Thus, increases in sphingomyelins and ceramides, while reductions in plasma sphingosine-1-phosphate (SIP), may contribute to cardiovascular diseases [18].

Exercise is an adjuvant therapy for many chronic diseases, including cardiovascular diseases associated with obesity [19]. Physical exercise leads to physiological changes in energy homeostasis, as it depends on changes in cellular responses to internal and external stress. Along these lines, some reports have observed that exercise can also affect human metabolism in plasma, which is the most responsive environment to changes in the body [20]. The effect of physical exercise on the lipid profile was notable in short-term interventions in postmenopausal women with dyslipidemia or obesity [21]. Another study showed that 8 weeks of combined physical training in women with obesity showed changes in different classes of lipids [22]. Furthermore, the physical activity was associated with lower TMAO levels, suggesting a possible new mechanism about physical activity as it protects cardio-metabolic health [23]. However, the effects of supervised exercise training on circulating TMAO and sphingolipid levels in subjects with obesity have not yet been reported.

Aerobic and strength training, when performed separately, have several benefits when it comes to obesity [24,25], but recent research demonstrates how the combination of both trainings (strength and aerobic) in the same session provides changes in levels ranging from strength gain to improvements in cardiometabolic and changes in epigenetic patterns in women undergoing combined physical training [26,27,28], achieving the benefits of aerobic and strength training combined in the same training session.

Therefore, we hypothesize that combined physical training is an effective tool to improve the metabolic system of women with obesity, protecting against cardiovascular diseases through reducing TMAO and plasma sphingolipid concentrations, regardless of weight loss. Therefore, we aimed to evaluate the effects of 8 weeks of combined physical training on plasma TMAO and sphingolipid levels in women with obesity.

## 2. Materials and Methods

### 2.1. Ethical Aspects, Participants, and Study Design

This prospective study was conducted in compliance with the Declaration of Helsinki. It was approved by the Research Ethics Committee of the University of the State of Minas Gerais, Divinópolis Unit (protocol 67644723.8.0000.5115) and registered in ClinicalTrials.gov (NTC 03119350). All subjects gave written consent for participation.

**Figure 1:**
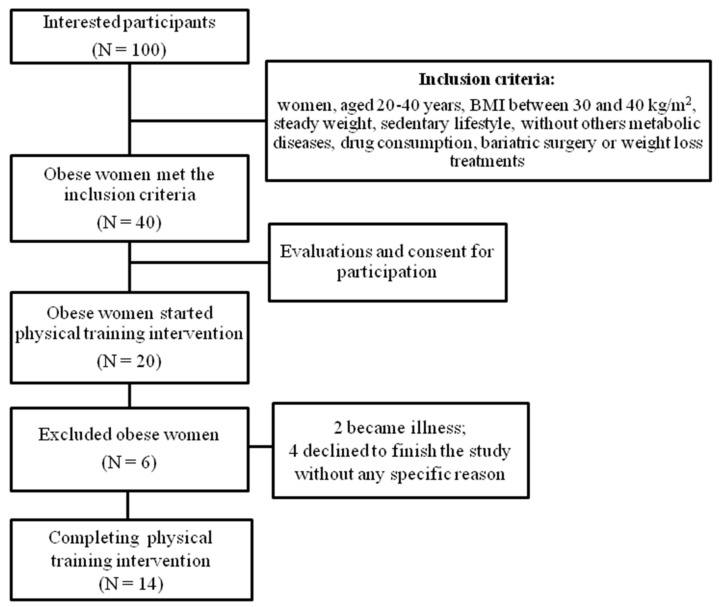
Flowchart.

After the study was released, websites and social networks advertised the protocol. Approximately 100 subjects were interested in participating in the study. However, the inclusion criteria were as follows: women aged 20–40 years, BMI between 30 and 40 kg/m^2^, steady weight, sedentary lifestyle, without other metabolic diseases, drug consumption, bariatric surgery, or weight loss treatments. The 40 obese women met the inclusion criteria; however, only 20 obese women started the intervention, and 14 obese women finished the intervention (Figure 1).

The intervention program lasted 12 weeks (two weeks of evaluation before and after the intervention, two weeks of physical exercise adaptation and physical test evaluation, and eight weeks of the physical training program). All subjects were clinically evaluated before and after the intervention of physical training.

### 2.2. Body Composition and Anthropometric Data Wall-Mounted Stadiometer

All participants were in an 8–10 h fasting state for evaluation. An electronic platform Fiziola™ scale with a precision of 0.1 kg and a maximum capacity of 300 kg measured body weight. A wall-mounted stadiometer with 0.5 cm graduation was used to measure body height. The body composition was evaluated by the deuterium oxide dilution method [29], each volunteer having received a dose of 1 mL/kg of 7% deuterium oxide (Cambridge Isotope, Cambridge, MA, USA). Urine samples were collected before and three hours after dose intake. Deuterium enrichment in urine samples was determined by mass spectrometry as previously reported (Europa Scientific Hydra System, Cheshire, UK) [30].

### 2.3. Plasma Collection and Biochemical Quantification

Blood samples were collected after 8–10 h fasting in heparin tubes, and plasma was separated by centrifugation. Creatinine, cholesterol, high-density lipoprotein cholesterol (HDL-c), and triglycerides were assayed by spectrophotometer (Labtest Diagnóstica S.A^®^, Lagoa Santa, Brazil).

TMAO and its precursors (betaine, choline, carnitine, and TMA) were analyzed by liquid chromatography tandem mass spectrometry (LC-MS/MS) using a Xevo^®^ TQD system with an electrospray ionization interface and an Acquity H-Class^®^ UPLC™ system (Waters Corporation, Milford, MA, USA). All solvents were of chromatographic grade and obtained from Biosolve^®^ (Valkenswaard, The Netherlands). Standards were purchased from Sigma Aldrich^®^ (Saint-Quentin Fallavier, France). A set of standard reference solutions was prepared and serially diluted in acetonitrile to obtain a curve of 7 standard solutions ranging from 0.05 to 10 µmol/L. From plasma samples (20 µL), TMAO and precursors were extracted with 180 µL of acetonitrile containing exogenous internal standards at 3 µmol/L (2H9-choline, 2H9-carnitine, 13C2-betaine, [13C3,15N]-TMA, and 2H9-TMAO). Samples were mixed and centrifuged for 10 min at 10,000× *g* (10 °C). Supernatants were transferred to small glass vials and analyzed by LC-MS/MS. From these samples, 10 µL (of TMAO and precursors) were injected onto a C18 HILIC^®^-BEH column (1.7 µm particle size, 2.1 mm internal diameter × 100 mm length, Waters Corporation^®^), maintained at 30 °C. Compounds were separated using a linear gradient of mobile phase B (98% acetonitrile, 0.1% formic acid, 1.9% MQ water) and mobile phase A (10 mmol/L ammonium acetate, 0.1% formic acid) at a flow rate of 400 µL/min. Mobile phase A was held constant for 1 min at 1%, linearly increased from 1% to 45% over 6.5 min, held at 45% for 0.5 min, and returned to the initial condition of 1% at 8.5 min, remaining constant for 2.5 min before the next injection. Target compounds were detected by mass spectrometer (LC-MS/MS) with electrospray ionization operating in positive ion mode (capillary voltage, 1.5 kV; desolvation gas flow (N2) and temperature, 650 L/h and 350 °C; source temperature, 150 °C). Multiple reaction monitoring modes were applied for MS/MS detection. Peak area ratios between unlabeled compounds and their respective internal standards constituted the detector response. Standard solutions were used to plot calibration curves for quantification. Linearity, expressed by mean r^2^ values greater than 0.998 for all compounds (linear regression, 1/x weighting, excluding the origin), was achieved. Intra- and inter-assay imprecisions were less than 9.7% for all compounds. Recoveries, assessed with internal standards, exceeded 96% [29]. It is important to note that endogenous TMA was not detected in most samples and hence is not documented here.

For the quantification of plasma sphingolipids, a set of standard reference solutions, including sphingosine-1-phosphate (S1P; d18:1), nine species of ceramide (Cer), and nine species of sphingomyelin (SM) (Avanti Polar Lipids, Alabaster, AL, USA), were prepared by serial dilution with methanol to obtain seven standard solutions, ranging from 1–500 nmol/L for Cer, 2–1000 nmol/L for S1P, and 0.04–20 mmol/L for SM. From plasma samples, 10 µL (of sphingolipids) were extracted with 500 µL of a methanol/chloroform mixture (2/1, *v*/*v*) containing exogenous internal standards [IS; Cer (d18:1/17:0) 500 nmol/L; S1P (d17:1) 500 nmol/L; and SM (d18:1/17:0) 5 µmol/L]. Samples were mixed and centrifuged for 10 min at 20,000× *g* (10 °C), and supernatants were dried under a stream of nitrogen gas and reconstituted in 100 µL of pure methanol for liquid chromatography tandem mass spectrometry (LC-MS/MS) analysis, performed on a Xevo TQD mass spectrometer with electrospray ionization interface and Acquity H-Class^®^ UPLC™ system (Waters Corporation, Milford, MA, USA). Data acquisition and analysis were conducted using MassLynx1 and TargetLynx1 software version 4.1, respectively (Waters Corporation, Milford, MA, USA). From these samples, 10 µL were injected onto an Acquity^®^ BEH C18 reverse-phase LC column, 2.1 Ø × 50 mm in length, where compounds were separated. The mobile phases were as follows: Phase A: 5/95 (acetonitrile/water *v*/*v*) + 0.1% formic acid + 10 mM ammonium formate solution, and Phase B: 50/50 (isopropanol/acetonitrile *v*/*v*) + 0.1% formic acid + 10 mM ammonium formate. The U-HPLC elution gradient coupled to MS was composed of the following: 60% Phase A at minute zero, decreasing to 1% at 4 min and maintained until 5.5 min, returning to 60% at 6 min and remaining until 8 min before the next analysis cycle. Sphingolipids were detected by MS with electrospray ionization operating in positive ion mode (capillary voltage, +3 kV; desolvation gas flow [N2] and temperature, 1000 L/h and 400 °C; source temperature, 150 °C). Multiple reaction monitoring (MRM) mode was applied. Linearity, expressed by mean r², was >0.998 for all compounds (linear regression, 1/x weighting, origin excluded). Intra- and inter-assay method imprecisions were evaluated in four separate experiments (six replicates for four concentrations), with RSDs < 13%, <6%, and <9% for Cer, SM, and S1P, respectively. Recoveries were assessed with IS and were >91% [31].

### 2.4. Physical Performance Test

Physical performance tests were performed before and after 8 weeks of intervention. The aerobic performance was evaluated by an adapted, incremental Shuttle walking test [32]. It required the participants to walk/run up and down a 10-meter course, which started at 4 km/h pf speed, increasing 0.28 m/s every 3 min by stage. The speed at which the participant walked/ran was dictated by an audio signal and was interrupted when subjects could not maintain the determined rhythm [33]. After each stage (3 min), 25 μL of blood was collected to determine the lactate threshold. Lactate was determined by an electrochemical lactometer (Yellow Springs™ Instruments model 1500 Sport). The fixed point was used for lactating the threshold (lactate threshold 1 = 2.0 mM; lactate threshold 2 = 3.5 mM). A curve was constructed between lactate thresholds and the test’s speed performed by the OriginPro versão 7.0 program (OriginLab Corporation^®^, Northampton, MA, USA). To determine which speed the participants were at the time of both lactate thresholds, the lactate threshold speed 1 (SpeedLT1) and lactate threshold speed 2 (SpeedLT2) were estimated. Finally, the estimated maximum oxygen consumption (VO2max) was determined, according to Heyward [34].

### 2.5. Physical Training Intervention

The intervention was executed in a Ribeirão Preto School Gym of the University of São Paulo, 100% supervised. The subjects who completed the intervention had a mean 80% of participation in total days of training. The combined physical training (alternating strength and aerobic exercise) consisted of 15 stations of strength exercises (for all the main muscle groups) for 30 s (at least ten repetitions per exercise) alternated with 30 s of jogging (between strength exercises). The strength exercises were performed in a circuit manner, alternating upper and lower limb exercises (flying chest, flexor, biceps, leg extension, straight abdominal, calf raise, bench press, leg press, front pull, infraumbilical abdominal, squat, press, lunge, triceps) with dumbbells and machines. The total circuit was repeated three times. The physical training intervention lasted eight weeks (with a frequency of 3 times/week with 55 min/day of duration and intensity of 75 to 90% of HRmax), but before accounting for this time, two weeks of adaptation to the exercise took place [35]. The intensity of training was controlled by the heart frequency meter (Polar^®^) and rating of perceived exertion (RPE), according to Foster [36]. The same-trained professional supervised all exercise sessions and the heart rate of participants. During the intervention, we emphasized to all participants to keep constant food intake. There were no diet intake restrictions.

### 2.6. Statistical Analyses

Descriptive statistics consisted of mean and standard deviation. After checking the normality of the sample (Shapiro–Wilk test). The paired t-tests were used for group comparison. The effect size was calculated by d-Cohen. Variation percentage was observed by differences between baseline and post-intervention by the following formula: fold-change % = ((Post − Pre)/Pre)x100); the percentage was presented in mean values. Results were considered significant at *p* ≤ 0.05. All analyses were performed by Jamovi 2.3 version software.

## 3. Results

After combined physical training intervention, no differences were observed for anthropometric data, except for waist circumference. However, there was an improvement in physical performance (Table 1). The fat-free mass and fat mass, respectively, increased by 4% and decreased by negative 4% (*p* > 0.05). The waist circumference also decreased after training (*p* < 0.05). The increased VO2max by 8%, SpeedLT1, and SpeedLT2 by 12% (*p* < 0.05). Just as we found an improvement in physical performance, in Table 2, it is observed that there was a reduction in TMAO (95%CI: Pre 4.91–12.12 vs. Post 3.47–6.80), independent of its precursors.

For the lipid profile, there was a reduction in total cholesterol (95%CI: Pre 168.0–188.2 vs. Post 156.3–177.3). When the analysis of sphingolipids was carried out, the physical training intervention caused the reduction of concentration of 10 lipids, including ceramides and sphingomyelin, with an increase in SIPd18:1 (Table 3).

## 4. Discussion

The 8 weeks of combined physical training were sufficient to promote protection against cardiovascular diseases through the reduction of TMAO, ceramides, and sphingomyelins. It also increases physical performance (VO2max, SpeedLT1, and SpeedLT2), independent of weight loss.

The study did not show a change in total body weight attributed to the duration of the training period since some studies have found weight loss only after ten weeks in obese/overweight women [38,39]. Despite subjects keeping constant weight, the results showed increased physical performance by enhancing VO2max, SpeedLT1, and SpeedLT2. The enhancements are also indicators of lower pathological risk factors, independent of weight loss [40]. In agreement with our data, Kong et al. [41] found similar results in women with obesity/overweight after five aerobic training weeks. There were also body metabolic changes with improved strength and aerobic performance that can prevent comorbidity associated with obesity [40]. Physical activity is associated with improvements in cardiometabolic health, with previously elucidated mechanisms including insulin resistance, lipid metabolism, and chronic low-grade inflammation [42,43].

It is important to consider that the women with obesity in this study had a normal lipid profile (except for HDL-C) and creatinine level within the references for the Brazilian population. The lipid profile is an essential biomarker of CVD. In recent years, numerous studies recognized the TMAO as an enhancer of cardiovascular risk via atherosclerotic lesion development [8,10,15].

Our study found values within Brazilian standards for standards considered normal for lipid profile, but a decreased level of plasma TMAO and total cholesterol after 8 weeks of combined physical training. Bordoni et al. [44] evaluated a group of older women, whether a 6-month l-leucine or l-leucine and l-carnitine supplementation combined with a resistance training protocol. No differences between groups at basal levels were observed for lipid profile and showed that l-carnitine supplementation increases TMAO level and no significant effects on TMAO were exerted by training alone. Creatinine is an important biomarker for assessing renal function, especially in people with obesity, due to kidney disease risk factors [45]. The values found in our study are within the normal range of 0.5 to 1.1 mg/dL [37]. No significant changes were demonstrated after physical training on plasma creatinine.

We found reductions in plasma concentrations of TMAO, a novel mechanism and clinical marker of risk factors for cardiovascular disease [10]. Usually, studies show that circulating levels of TMAO are sensitive to change through manipulation of dietary [46] and that Erickson et al. [47] showed that changed TMAO levels after exercise and hypocaloric diet. Nevertheless, the effects of exercise on TMAO remain controversial, as reports showed decreased [48], increased [49], or unmodified [50] responses after physical training. However, an animal study showed that voluntary exercise could inhibit elevations of gut macrobiotic-dependent metabolite TMAO [19]. In these studies, plasma TMAO concentrations were lower, leading to some biases in data interpretation [50]. The systemic concentration of TMAO in an individual’s normal weight is between 0.5 µmol and 5.0 µmol [51,52]. Concentrations between 6.0 and 8.0 µmol are usually found in people with heart failure [53]. In our study, the TMAO concentration found was 8.2 ± 6.4 µmol to 5.4 ± 2.8 µmol, changing to normal levels after the exercise intervention.

Normally, TMAO is produced by the intestinal microbiota [54]. While the microbiota profile can be influenced by physical activity, particularly in obese adults [55,56]. In the same sense, the body composition can be related to TMAO concentrations due to a positive association between the fat-free mass, visceral fat, and plasma choline and carnitine [46]. Another study showed that TMAO was positively associated with adiposity [9].

Other variables analyzed and related to adiposity, ceramides, and sphingomyelins were also reduced after the physical training intervention. The elevation of ceramides in plasma has been related to obesity, increased insulin resistance, and a more significant occurrence of cardiovascular diseases [57]. Specifically, ceramides (Cer 16:0 and Cer 18:0) have been described as increased in people with obesity and may be negatively associated with insulin sensitivity and body energy expenditure [58,59,60]. In the same way, ceramide levels in plasma were correlated with higher BMI values [61,62]. High levels of ceramides are also associated with increased hunger and weight gain, as they mediate ghrelin and leptin signaling in the hypothalamus [63].

Studies have shown that ceramide levels are responsive to physical exercise interventions, analyzed in plasma, adipose tissue, or skeletal muscle [64,65,66]. Corroborating our findings, we found reductions in ceramides Cer 16:0, Cer 18:0, and Cer 22:0, considered the most abundant ceramides in plasma [64]. Meanwhile, the only lipid that increased after physical training was sphingosine-1-phosphate (S1P), following the same direction as the study by Baranowski et al. [67], which shows an increase in S1P in the plasma of 20 healthy men after aerobic physical training sessions. The same result was found by Ksiazek et al. [68] in 30 healthy men after eight weeks of aerobic physical training. It is believed that S1P is linked to high-density lipoprotein (HDL) and albumin. Thus, its increase may be a mechanism with cardioprotective properties and is related to the benefits of the effects of physical exercise on cardiovascular diseases [69]. Our data suggest that Cer16:0, Cer 18:0, Cer22:0, and S1P could indicate the presence or absence of cardiovascular risk.

Ceramides also play an essential role in the metabolism of sphingolipids, which can be converted into sphingomyelin (SM), which, following liver synthesis, is incorporated into low-density lipoprotein (VLDL) [70]. Typically, sphingomyelins are accumulated in human atherosclerotic plaques since low-density lipoprotein (LDL) is present in atherosclerotic plaques and has high sphingomyelin levels [71]. Athletes have a lower concentration of sphingomyelin when compared to obese people and people with type 2 diabetes mellitus [72]. Our study showed a reduction in ceramides, sphingomyelins, and total cholesterol after the intervention with physical training.

This study describes a significant reduction of TMAO concentrations and sphingolipid markers and an improvement in physical performance. These results are essential in protecting cardiometabolic health. We hypothesized that these findings were consistent. However, our study has some limitations, such as the low number of subjects, the short time duration of physical training, and the indirect VO_2_max measure, and they deserve to be confirmed in further studies (with the control group). Moreover, we did not monitor the energy intake and diet of participants during follow-up. However, we reinforced it to all participants to keep constant dietary intake habits. Usually, the studies that evaluated the effects of exercise on plasma TMAO concentrations only gave recommendations to increase physical activity levels or change the diet intake. Our study’s advantage is the highly controlled supervised intervention on exercise, including frequency (days per week), duration of each exercise session, and training intensity.

## 5. Conclusions

The combined physical training reduced TMAO, sphingomyelin, and ceramide concentrations, while physical performance increased independent of weight loss. It suggests that physical exercise could participate in cardiovascular risk protection in women with obesity.

TMAO is an independent risk factor for numerous metabolic diseases, while physical exercise is a protective factor. This study concluded that 8 weeks of combined physical training in women with obesity promoted improved metabolic health and protection against other diseases, regardless of weight loss or dietary intervention.

## Figures and Tables

**Table 1:** Anthropometric, body composition, and performance data before and after physical training intervention from women with obesity.

Variables	Pre	Post	*p*-Value	d-Cohen
BMI (kg/m^2^) *	32 ± 2	33 ± 2	0.169	−0.389
Weight (kg) *	86 ± 8	87 ± 9	0.150	−0.408
%FM	47 ± 3	45 ± 5	0.312	0.281
%FFM	53 ± 3	55 ± 5	0.312	−0.281
Waist circumference *	93 ± 2	91 ± 2	**0.027**	0.664
Hip circumference	118 ± 7	117 ± 7	0.702	0.105
Waist/Hip rate	0.79 ± 0.08	0.77 ± 0.06	**0.001**	1.104
VO2max (ml/kg/min) *	35 ± 3	38 ± 3	**0.002**	−1.015
Speed_LT1_ (km/h)	5 ± 1	6 ± 1	**0.001**	−1.134
Speed_LT2_ (km/h)	6 ± 1	7 ± 1	**<0.001**	−1.483

Note—Data are expressed as means ± standard deviations (M ± SD). Paired T test. *p* ≤ 0.05. d-Cohen represents effect size. Bold represent significant *p*-values. Abbreviations: BMI, body mass index; FM, fat mass; FFM, fat-free mass; VO2max, maximum oxygen consumption; SpeedLT1, the speed of lactate threshold 1; SpeedLT2, the speed of lactate threshold 2. * Published data [32,34].

**Table 2:** Biochemicals, TMAO, and precursors data before and after physical training intervention from women with obesity.

Variables	Pre	Post	*p*-Value	d-Cohen	Reference Value
Creatinine (mg/dL)	0.83 ± 0.9	0.84 ± 0.9	0.850	−0.051	0.6 and 1.2
Cholesterol (mg/dL)	177.1 ± 17.5	166.8 ± 18.2	**0.049**	0.581	<190
HDL-c (mg/dL)	29.8 ± 6.2	31.9 ± 10.2	0.281	−0.300	>40
LDL-c (mg/dL)	126.1 ± 20.6	115.4 ± 18.9	0.095	0.481	<130
Triglycerides (mg/dL)	110.8 ± 56.4	98.1 ± 49.2	0.143	0.416	<150
TMAO (µmol)	8.5 ± 6.2	5.1 ± 2.8	**0.017**	0.730	-
Choline (µmol)	1.7 ± 0.3	1.9 ± 0.4	0.235	−0.333	-
Betaine (µmol)	31.7 ± 7.5	32.3 ± 11.1	0.768	−0.080	-
Carnitine (µmol)	41.3 ± 7.5	41.0 ± 7.2	0.944	0.054	-

Note—Data are expressed as means ± standard deviations (M ± SD). Paired T test. *p* ≤ 0.05. d-Cohen represents effect size. Bold represent significant *p*-values. Abbreviations: HDL-c, high-density lipoprotein cholesterol; LDL-c, low-density lipoprotein cholesterol; TMAO, trimethylamine N-oxide, reference value [37]. There are no reference values for TMAO and its precursors.

**Table 3:** Sphingolipid concentrations in plasma before and after physical training intervention from women with obesity.

Lipids	Pre (nmol/L)	Post (nmol/L)	*p*-Value	d-Cohen
S1P d18:1	366.27 ± 82.77	471.214 ± 75.87	**0.003**	−0.974
CER 16:0	201.88 ± 36.83	160.10 ± 50.19	**0.028**	0.659
CER 18:0	133.04 ± 60.77	104.79 ± 42.11	**0.044**	0.597
CER 20:0	14.87 ± 6.97	13.58 ± 4.62	0.498	0.194
CER 22:0	1909.23 ± 411.61	1421.06 ± 375.39	**0.015**	0.753
CER 24:0	265.79 ± 45.14	214.64 ± 67.71	0.072	0.523
CER 18:1	9.50 ± 2.38	7.81 ± 2.24	0.112	0.455
CER 20:1	7.83 ± 2.18	6.38 ±2.74	0.120	0.444
CER 22:1	7.18 ± 3.46	6.65 ± 2.45	0.588	0.148
CER 24:1	1098.98 ± 340.48	956.50 ± 370.26	0.329	0.271
SM 16:0	54.64 ± 5.47	47.96 ± 9.05	**0.024**	0.681
SM 18:0	9.35 ± 2.64	7.37 ± 2.35	**0.025**	0.675
SM 20:0	18.34 ± 3.09	16.22 ± 3.90	**0.059**	0.553
SM 22:0	19.77 ± 2.95	17.01 ± 4.45	**0.025**	0.677
SM 24:0	20.44 ± 2.88	15.16 ± 5.63	**0.003**	0.963
SM 18:1	3.35 ± 0.81	2.80 ± 0.85	**0.043**	0.598
SM 20:1	1.19 ± 0.22	0.99 ± 0.282	**0.047**	0.586
SM 22:1	19.66 ± 2.99	18.50 ± 4.66	0.333	0.268
SM 24:1	34.49 ± 6.74	33.36 ± 9.48	0.682	0.112

Note—Data are expressed as means ± standard deviations (M ± SD). Paired T test. *p* ≤ 0.05. d-Cohen represents effect size. Bold represent significant *p*-values. Abbreviations: S1P, sphingosine-1-phosphate; CER, ceramides; SM, sphingomyelin.

## Data Availability

The original contributions presented in the study are included in the article/Supplementary Material, further inquiries can be directed to the corresponding author/s.

## Supplementary Materials

The following supporting information can be downloaded at: https://www.mdpi.com/article/10.3390/metabo14080398/s1.

## References

[1] Dai H., Alsalhe T.A., Chalghaf N., Riccò M., Bragazzi N.L., Wu J. (2020). The global burden of disease attributable to high body mass index in 195 countries and territories, 1990–2017: An analysis of the Global Burden of Disease Study. PLoS Med..

[2] Romieu I., Dossus L., Barquera S., Blottière H.M., Franks P.W., Gunter M., Hwalla N., Hursting S.D., Leitzmann M., Margetts B. (2017). IARC working group on Energy Balance and Obesity. Energy balance and obesity: What are the main drivers?. Cancer Causes Control..

[3] Panuganti K.K., Nguyen M., Kshirsagar R.K. (2024). Obesity. StatPearls.

[4] Sturm R., An R. (2014). Obesity and economic environments. CA Cancer J. Clin..

[5] Thaker V.V. (2017). Genetic and epigenetic causes of obesity. Adolesc. Med. State Art Rev..

[6] Kesherwani V., Chavali V., Hackfort B.T., Tyagi S.C., Mishra P.K. (2015). Exercise ameliorates high fat diet induced cardiac dysfunction by increasing interleukin 10. Front. Physiol..

[7] Russo C., Jin Z., Homma S., Rundek T., Elkind M.S., Sacco R.L., Di Tullio M.R. (2011). Effect of obesity and overweight on left ventricular diastolic function: A community-based study in an elderly cohort. J. Am. Coll. Cardiol..

[8] Randrianarisoa E., Lehn-Stefan A., Wang X., Hoene M., Peter A., Heinzmann S.S., Zhao X., Königsrainer I., Königsrainer A., Balletshofer B. (2016). Relationship of Serum Trimethylamine N-Oxide (TMAO) Levels with early Atherosclerosis in Humans. Sci. Rep..

[9] Heianza Y., Sun D., Smith S.R., Bray G.A., Sacks F.M., Qi L. (2018). Changes in Gut Microbiota-Related Metabolites and Long-term Successful Weight Loss in Response to Weight-Loss Diets: The POUNDS Lost Trial. Diabetes Care.

[10] Cho C.E., Caudill M.A. (2017). Trimethylamine-N-Oxide: Friend, Foe, or Simply Caught in the Cross-Fire?. Trends Endocrinol. Metab..

[11] Missailidis C., Hällqvist J., Qureshi A.R., Barany P., Heimbürger O., Lindholm B., Stenvinkel P., Bergman P. (2016). Serum Trimethylamine-N-Oxide Is Strongly Related to Renal Function and Predicts Outcome in Chronic Kidney Disease. PLoS ONE.

[12] Rohrmann S., Linseisen J., Allenspach M., von Eckardstein A., Müller D. (2016). Plasma Concentrations of Trimethylamine-N-oxide Are Directly Associated with Dairy Food Consumption and Low-Grade Inflammation in a German Adult Population. J. Nutr..

[13] Li X.S., Obeid S., Klingenberg R., Gencer B., Mach F., Räber L., Windecker S., Rodondi N., Nanchen D., Muller O. (2017). Gut microbiota-dependent trimethylamine N-oxide in acute coronary syndromes: A prognostic marker for incident cardiovascular events beyond traditional risk factors. Eur. Heart J..

[14] Qi J., You T., Li J., Pan T., Xiang L., Han Y., Zhu L. (2018). Circulating trimethylamine N-oxide and the risk of cardiovascular diseases: A systematic review and meta-analysis of 11 prospective cohort studies. J. Cell Mol. Med..

[15] Heianza Y., Ma W., Manson J.E., Rexrode K.M., Qi L. (2017). Gut Microbiota Metabolites and Risk of Major Adverse Cardiovascular Disease Events and Death: A Systematic Review and Meta-Analysis of Prospective Studies. J. Am. Heart Assoc..

[16] Mathis D. (2013). Immunological goings-on in visceral adipose tissue. Cell Metab..

[17] Bikman B.T., Summers S.A. (2011). Ceramides as modulators of cellular and whole-body metabolism. J. Clin. Invest..

[18] Iqbal J., Walsh M.T., Hammad S.M., Hussain M.M. (2017). Sphingolipids and Lipoproteins in Health and Metabolic Disorders. Trends Endocrinol. Metab..

[19] Zhang H., Meng J., Yu H. (2017). Trimethylamine N-oxide Supplementation Abolishes the Cardioprotective Effects of Voluntary Exercise in Mice Fed a Western Diet. Front. Physiol..

[20] Latino F., Cataldi S., Carvutto R., De Candia M., D’Elia F., Patti A., Bonavolontà V., Fischetti F. (2021). The Importance of Lipidomic Approach for Mapping and Exploring the Molecular Networks Underlying Physical Exercise: A Systematic Review. Int. J. Mol. Sci..

[21] He M., Hu S., Wang J., Wang J., Găman M.A., Hariri Z., Tian Y. (2023). Effect of resistance training on lipid profile in postmenopausal women: A systematic review and meta-analysis of randomized controlled trials. Eur. J. Obs. Obstet. Gynecol. Reprod. Biol..

[22] San Martin R., Brandao C.F.C., Junqueira-Franco M.V.M., Junqueira G.P., de Freitas E.C., de Carvalho F.G., Rodrigues C.H.P., Aguesse A., Billon-Crossouard S., Krempf M. (2022). Untargeted lipidomic analysis of plasma from obese women submitted to combined physical exercise. Sci. Rep..

[23] Argyridou S., Bernieh D., Henson J., Edwardson C.L., Davies M.J., Khunti K., Suzuki T., Yates T. (2020). Associations between physical activity and trimethylamine N-oxide in those at risk of type 2 diabetes. BMJ Open Diabetes Res. Care.

[24] Oh D.H., Lee J.K. (2023). Effect of Different Intensities of Aerobic Exercise Combined with Resistance Exercise on Body Fat, Lipid Profiles, and Adipokines in Middle-Aged Women with Obesity. Int. J. Environ. Res. Public Health.

[25] Mezghani N., Ammar A., Boukhris O., Abid R., Hadadi A., Alzahrani T.M., Trabelsi O., Boujelbane M.A., Masmoudi L., Ouergui I. (2022). The Impact of Exercise Training Intensity on Physiological Adaptations and Insulin Resistance in Women with Abdominal Obesity. Healthcare.

[26] Rodrigues G.D.S., Rodrigues K.P., de Almeida M.L., Sobrinho A.C.D.S., Noronha N.Y., Benjamim C.J.R., Silva S.D., Rodrigues J.A.L., Júnior C.R.B. (2023). Comparing Fourteen Weeks of Multicomponent Training Versus Combined Training in Physically Inactive Older Women: A Randomized Trial. Int. J. Environ. Res. Public Health.

[27] Yumi Noronha N., da Silva Rodrigues G., Harumi Yonehara Noma I., Fernanda Cunha Brandao C., Pereira Rodrigues K., Colello Bruno A., Sae-Lee C., Moriguchi Watanabe L., Augusta de Souza Pinhel M., Mello Schineider I. (2022). 14-weeks combined exercise epigenetically modulated 118 genes of menopausal women with prediabetes. Front. Endocrinol..

[28] da Silva Rodrigues G., Noronha N.Y., Almeida M.L., Sobrinho A.C.D.S., Watanabe L.M., Pinhel M.A.S., de Lima J.G.R., Zhang R., Nonino C.B., Alves C.R.R. (2023). Exercise training modifies the whole blood DNA methylation profile in middle-aged and older women. J. Appl. Physiol..

[29] Resende C.M., Camelo Júnior J.S., Vieira M.N., Ferriolli E., Pfrimer K., Perdoná G.S., Monteiro J.P. (2011). Body composition measures of obese adolescents by the deuterium oxide dilution method and by bioelectrical impedance. Braz. J. Med. Biol. Res..

[30] Trenteseaux C., Gaston A.T., Aguesse A., Poupeau G., de Coppet P., Andriantsitohaina R., Laschet J., Amarger V., Krempf M., Nobecourt-Dupuy E. (2017). Perinatal Hypercholesterolemia Exacerbates Atherosclerosis Lesions in Offspring by Altering Metabolism of Trimethylamine-N-Oxide and Bile Acids. Arter. Arterioscler. Thromb. Vasc. Biol..

[31] Croyal M., Kaabia Z., León L., Ramin-Mangata S., Baty T., Fall F., Billon-Crossouard S., Aguesse A., Hollstein T., Sullivan D.R. (2018). Fenofibrate decreases plasma ceramide in type 2 diabetes patients: A novel marker of CVD?. Diabetes Metab..

[32] Singh S.J., Morgan M.D., Scott S., Walters D., Hardman A.E. (1992). Development of a shuttle walking test of disability in patients with chronic airways obstruction. Thorax.

[33] Brandao C.F.C., Nonino C.B., de Carvalho F.G., Nicoletti C.F., Noronha N.Y., San Martin R., de Freitas E.C., Junqueira-Franco M.V.M., Marchini J.S. (2020). The effects of short-term combined exercise training on telomere length in obese women: A prospective, interventional study. Sports Med. Open.

[34] Heyward V.H. (1992). Advanced fitness assessment and exercise prescription. Med. Sci. Sports Exerc..

[35] Brandao C.F.C., de Carvalho F.G., Souza A.O., Junqueira-Franco M.V.M., Batitucci G., Couto-Lima C.A., Fett C.A., Papoti M., Freitas E.C., Alberici L.C. (2019). Physical training, UCP1 expression, mitochondrial density, and coupling in adipose tissue from women with obesity. Scand. J. Med. Sci. Sports.

[36] Foster C. (1998). Monitoring training in athletes with reference to overtraining syndrome. Med. Sci. Sports Exerc..

[37] Szwarcwald C.L., Malta D.C., Pereira C.A., Figueiredo A.W., Almeida W.D.S., Machado I.E., Bacal N.S., Silva A.G.D., Silva Júnior J.B.D., Rosenfeld L.G. (2019). Reference values for laboratory tests of cholesterol, glycosylated hemoglobin and creatinine of the Brazilian adult population. Rev. Bras. Epidemiol..

[38] Ferreira F.C., Bertucci D.R., Barbosa M.R., Nunes J.E., Botero J.P., Rodrigues M.F., Shiguemoto G.E., Santoro V., Verzola A.C., Nonaka R.O. (2017). Circuit resistance training in women with normal weight obesity syndrome: Body composition, cardiometabolic and echocardiographic parameters, and cardiovascular and skeletal muscle fitness. J. Sports Med. Phys. Fitness.

[39] Tan S., Wang J., Cao L., Guo Z., Wang Y. (2016). Positive effect of exercise training at maximal fat oxidation intensity on body composition and lipid metabolism in overweight middle-aged women. Clin. Physiol. Funct. Imaging.

[40] Kennedy A.B., Lavie C.J., Blair S.N. (2018). Fitness or Fatness: Which Is More Important?. JAMA.

[41] Kong Z., Sun S., Liu M., Shi Q. (2016). Short-Term High-Intensity Interval Training on Body Composition and Blood Glucose in Overweight and Obese Young Women. J. Diabetes Res..

[42] Taylor R.S., Brown A., Ebrahim S., Jolliffe J., Noorani H., Rees K., Skidmore B., Stone J.A., Thompson D.R., Oldridge N. (2004). Exercise-based rehabilitation for patients with coronary heart disease: Systematic review and meta-analysis of randomized controlled trials. Am. J. Med..

[43] Warburton D.E., Nicol C.W., Bredin S.S. (2006). Health benefits of physical activity: The evidence. CMAJ.

[44] Bordoni L., Sawicka A.K., Szarmach A., Winklewski P.J., Olek R.A., Gabbianelli R. (2020). A Pilot Study on the Effects of l-Carnitine and Trimethylamine-N-Oxide on Platelet Mitochondrial DNA Methylation and CVD Biomarkers in Aged Women. Int. J. Mol. Sci..

[45] Vittori L.N., Romasco J., Tarozzi A., Latessa P.M. (2021). Urinary Markers and Chronic Effect of Physical Exercise. Methods Mol. Biol..

[46] Trøseid M., Hov J.R., Nestvold T.K., Thoresen H., Berge R.K., Svardal A., Lappegård K.T. (2016). Major Increase in Microbiota-Dependent Proatherogenic Metabolite TMAO One Year After Bariatric Surgery. Metab. Syndr. Relat. Disord..

[47] Erickson M.L., Malin S.K., Wang Z., Brown J.M., Hazen S.L., Kirwan J.P. (2019). Effects of Lifestyle Intervention on Plasma Trimethylamine N-Oxide in Obese Adults. Nutrients.

[48] Pechlivanis A., Kostidis S., Saraslanidis P., Petridou A., Tsalis G., Mougios V., Gika H.G., Mikros E., Theodoridis G.A. (2010). (1)H NMR-based metabonomic investigation of the effect of two different exercise sessions on the metabolic fingerprint of human urine. J. Proteome Res..

[49] Wang F., Han J., He Q., Geng Z., Deng Z., Qiao D. (2015). Applying (1)H NMR Spectroscopy to Detect Changes in the Urinary Metabolite Levels of Chinese Half-Pipe Snowboarders after Different Exercises. J. Anal. Methods Chem..

[50] Enea C., Seguin F., Petitpas-Mulliez J., Boildieu N., Boisseau N., Delpech N., Diaz V., Eugène M., Dugué B. (2010). (1)H NMR-based metabolomics approach for exploring urinary metabolome modifications after acute and chronic physical exercise. Anal. Bioanal. Chem..

[51] Ufnal M., Zadlo A., Ostaszewski R. (2015). TMAO: A small molecule of great expectations. Nutrition.

[52] Wang Z., Klipfell E., Bennett B.J., Koeth R., Levison B.S., Dugar B., Feldstein A.E., Britt E.B., Fu X., Chung Y.M. (2011). Gut flora metabolism of phosphatidylcholine promotes cardiovascular disease. Nature.

[53] Tang W.H., Wang Z., Fan Y., Levison B., Hazen J.E., Donahue L.M., Wu Y., Hazen S.L. (2014). Prognostic value of elevated levels of intestinal microbe-generated metabolite trimethylamine-N-oxide in patients with heart failure: Refining the gut hypothesis. J. Am. Coll. Cardiol..

[54] Koeth R.A., Wang Z., Levison B.S., Buffa J.A., Org E., Sheehy B.T., Britt E.B., Fu X., Wu Y., Li L. (2013). Intestinal microbiota metabolism of L-carnitine, a nutrient in red meat, promotes atherosclerosis. Nat. Med..

[55] Denou E., Marcinko K., Surette M.G., Steinberg G.R., Schertzer J.D. (2016). High-intensity exercise training increases the diversity and metabolic capacity of the mouse distal gut microbiota during diet-induced obesity. Am. J. Physiol. Endocrinol. Metab..

[56] Allen J.M., Mailing L.J., Niemiro G.M., Moore R., Cook M.D., White B.A., Holscher H.D., Woods J.A. (2018). Exercise Alters Gut Microbiota Composition and Function in Lean and Obese Humans. Med. Sci. Sports Exerc..

[57] Chavez J.A., Summers S.A. (2012). A ceramide-centric view of insulin resistance. Cell Metab..

[58] Turpin S.M., Nicholls H.T., Willmes D.M., Mourier A., Brodesser S., Wunderlich C.M., Mauer J., Xu E., Hammerschmidt P., Brönneke H.S. (2014). Obesity-induced CerS6-dependent C16:0 ceramide production promotes weight gain and glucose intolerance. Cell Metab..

[59] Raichur S., Wang S.T., Chan P.W., Li Y., Ching J., Chaurasia B., Dogra S., Öhman M.K., Takeda K., Sugii S. (2014). CerS2 haploinsufficiency inhibits β-oxidation and confers susceptibility to diet-induced steatohepatitis and insulin resistance. Cell Metab..

[60] Bajpeyi S., Myrland C.K., Covington J.D., Obanda D., Cefalu W.T., Smith S.R., Rustan A.C., Ravussin E. (2014). Lipid in skeletal muscle myotubes is associated to the donors’ insulin sensitivity and physical activity phenotypes. Obesity.

[61] Weir J.M., Wong G., Barlow C.K., Greeve M.A., Kowalczyk A., Almasy L., Comuzzie A.G., Mahaney M.C., Jowett J.B., Shaw J. (2013). Plasma lipid profiling in a large population-based cohort. J. Lipid Res..

[62] Mamtani M., Meikle P.J., Kulkarni H., Weir J.M., Barlow C.K., Jowett J.B., Bellis C., Dyer T.D., Almasy L., Mahaney M.C. (2014). Plasma dihydroceramide species associate with waist circumference in Mexican American families. Obesity.

[63] Ramírez S., Martins L., Jacas J., Carrasco P., Pozo M., Clotet J., Serra D., Hegardt F.G., Diéguez C., López M. (2013). Hypothalamic ceramide levels regulated by CPT1C mediate the orexigenic effect of ghrelin. Diabetes.

[64] Kasumov T., Li L., Li M., Gulshan K., Kirwan J.P., Liu X., Previs S., Willard B., Smith J.D., McCullough A. (2015). Ceramide as a mediator of non-alcoholic Fatty liver disease and associated atherosclerosis. PLoS ONE.

[65] Solomon T.P., Sistrun S.N., Krishnan R.K., Del Aguila L.F., Marchetti C.M., O’Carroll S.M., O’Leary V.B., Kirwan J.P. (2008). Exercise and diet enhance fat oxidation and reduce insulin resistance in older obese adults. J. Appl. Physiol..

[66] Amati F., Dubé J.J., Alvarez-Carnero E., Edreira M.M., Chomentowski P., Coen P.M., Switzer G.E., Bickel P.E., Stefanovic-Racic M., Toledo F.G. (2011). Skeletal muscle triglycerides, diacylglycerols, and ceramides in insulin resistance: Another paradox in endurance-trained athletes?. Diabetes.

[67] Baranowski M., Charmas M., Długołęcka B., Górski J. (2011). Exercise increases plasma levels of sphingoid base-1 phosphates in humans. Acta Physiol..

[68] Książek M., Charmas M., Klusiewicz A., Zabielski P., Długołęcka B., Chabowski A., Baranowski M. (2018). Endurance training selectively increases high-density lipoprotein-bound sphingosine-1-phosphate in the plasma. Scand. J. Med. Sci. Sports.

[69] Hoofnagle A.N., Vaisar T., Mitra P., Chait A. (2010). HDL lipids and insulin resistance. Curr. Diab. Rep..

[70] Ooi E.M., Watts G.F., Chan D.C., Chen M.M., Nestel P.J., Sviridov D., Barrett P.H. (2008). Dose-dependent effect of rosuvastatin on VLDL-apolipoprotein C-III kinetics in the metabolic syndrome. Diabetes Care.

[71] Kang S.C., Kim B.R., Lee S.Y., Park T.S. (2013). Sphingolipid metabolism and obesity-induced inflammation. Front. Endocrinol..

[72] Bergman B.C., Brozinick J.T., Strauss A., Bacon S., Kerege A., Bui H.H., Sanders P., Siddall P., Kuo M.S., Perreault L. (2015). Serum sphingolipids: Relationships to insulin sensitivity and changes with exercise in humans. Am. J. Physiol. Endocrinol. Metab..

