# Supplementary material for: Sphingolipid and Trimethylamine-N-Oxide (TMAO) Levels in Women with Obesity after Combined Physical Training
Source: Metabolites. 2024 Jul 23;14(8):398. doi: 10.3390/metabo14080398 (PMC11355985; doi:10.3390/metabo14080398)
Supplement: Supplementary file 1 [file metabolites-14-00398-s001.zip › metabolites-3062729-supplementary material.pdf]

Supplementary Material

Raw data of main results:

Trimethylamine N-oxide and its precursors

TMAO		Choline		Betaine		Carnitine	
Pre	Post	Pre	Post	Pre	Post	Pre	Post
4.3034	3.837	1.6415	1.7225	39.4702	20.4743	42.1953	34.3405
3.5281	3.0609	1.4052	1.5726	31.2542	38.9149	40.2251	35.6107
13.0925	12.541	1.5684	1.852	45.9142	56.2743	53.9224	49.8247
4.7107	3.0546	1.5606	1.6551	30.2668	29.7669	58.3133	58.4049
7.2363	6.3846	2.0924	1.2754	19.97	22.2297	31.9903	28.4446
22.9341	7.9252	1.7497	1.9699	19.227	19.1954	41.9612	39.6236
10.9667	4.5678	1.468	1.8424	36.142	38.4105	39.0534	39.1254
20.0282	8.7997	2.2632	2.0921	27.9326	27.4177	44.0381	40.3157
5.4171	5.3841	1.8546	2.4643	16.0221	16.9879	39.1425	43.635
4.0445	1.9774	1.5413	2.2312	29.9082	41.36	31.1952	43.2829
9.1268	4.0194	1.8454	2.2	39.3504	32.7617	43.553	37.7323
4.7265	3.4705	1.7374	2.5003	40.7445	34.9034	34.6216	39.3112
6.0589	2.967	2.2228	1.3843	40.9007	45.5884	43.5668	47.344
3.039	3.8906	1.697	2.1683	27.0891	28.5721	35.3578	38.0209

Sphingolipids:

S1P d18:1	
Pre	Post
283.726	329.686
492.5259	479.7351
231.6035	477.7184
396.3336	363.001
301.3794	491.2313
345.9913	412.0379
460.7253	481.5672
340.7769	468.7447
368.0129	618.4483
517.8638	496.4664
316.1785	544.5693
381.2086	391.743
402.7924	519.4605
288.6235	522.5809
366.2673	471.2136
82.77196	75.8792

CER 16:0		CER 18:0		CER 20:0		CER 22:0		CER 24:0		CER 18:1		CER 20:1		CER 22:1		CER 24:1	
Pre	Post	Pre	Post	Pre	Post	Pre	Post	Pre	Post	Pre	Post	Pre	Post	Pre	Post	Pre	Post
215.5222	173.3465	197.5911	156.4375	20.8026	20.4319	1728.731	1700.774	230.2924	210.3346	10.782	7.6898	7.3768	6.4513	8.1243	5.5054	1082.8	1088.77
201.3548	249.6655	145.7185	151.2128	20.1313	19.9445	1794.848	1560.181	267.9428	222.4794	8.7809	11.7675	6.963	4.8279	5.709	8.6157	981.8654	1152.146
283.6163	100.9265	94.6339	39.9143	12.55		1831.615	547.67	309.159	60.433	12.9717	3.9225	7.8788	1.4785	5.8413	0.6254	1163.767	305.6594
213.489	242.2402	82.4699	98.2007	6.584	11.7903	1894.502	1645.18	244.2039	303.0738	6.2917	5.7871	3.5448	4.3908	3.4455	5.9224	982.0906	1193.563
200.1185	231.6729	193.1062	159.1397	30.0787	14.3645	2289.365	1653.815	325.3244	216.7492	13.6114	8.7956	10.2631	11.2678	15.9694	10.2157	1960.321	1404.441
197.5654	150.4303	85.4925	57.7779	13.366	5.851	1466.317	1238.07	243.1378	205.3066	8.0342	8.2388	8.9914	4.3393	7.843	4.7593	1033.499	582.9589
168.806	181.5278	102.7019	145.5113	10.0177	19.0404	1310.019	2231.114	184.1523	340.0693	8.023	9.4391	7.3115	9.8126	2.7043	8.2888	748.2846	1731.824
210.9991	145.0502	74.3955	79.6528	6.849	12.9522	1827.031	1524.067	287.5785	241.424	5.5343	8.3946	9.8249	5.4585	7.0295	7.1165	685.8643	790.6344
108.7651	109.7727	57.0604	81.5778	6.3161	15.2028	1455.44	1528.22	211.7217	267.9062	9.9401	11.0009	6.0465	10.2394	2.9273	6.9475	569.0621	1193.723
197.7206	125.8659	121.9379	102.8933	14.1191	13.3237	2597.415	1237.728	250.4415	133.0539	10.5229	4.2831	11.974	7.6073	9.3157	7.8388	1058.581	839.6532
215.185	142.2702	264.2215	164.7782	24.5497	15.6848	2444.956	1214.334	283.7756	210.3988	7.9869	8.4458	7.6394	7.4847	10.6298	8.9843	1370.237	778.8041
196.4332	117.9432	103.6008	73.9319	13.021	10.6475	1717.612	1255.197	284.1835	211.8139	8.5278	8.5484	6.5274	7.1609	6.0413	8.6315	1202.297	1005.203
223.4832	109.1378	206.9205	76.6891	16.0762	6.2964	1806.252	1354.768	246.7011	171.6409	12.3141	6.5095	9.798	3.7981	8.7992	5.2793	1335.643	674.5504
193.3247	161.5689	132.7562	79.384	13.7659	11.0893	2565.179	1203.833	352.3947	210.283	9.6848	6.6266	5.5325	5.017	6.1713	4.4928	1211.443	649.0839
201.8845	160.1013	133.0433	104.793	14.87338	13.5861	1909.234	1421.068	265.7864	214.6405	9.500414	7.817807	7.833721	6.381007	7.182207	6.658814	1098.983	956.5009
36.83123	50.18965	60.77579	42.11457	6.971852	4.620644	411.611	375.3988	45.1442	67.71504	2.384162	2.247961	2.185225	2.743356	3.464384	2.45889	340.4797	370.2663

SM 16:0		SM 18:0		SM 20:0		SM 22:0		SM 24:0		SM 18:1		SM 20:1		SM 22:1		SM 24:1	
Pre	Post	Pre	Post	Pre	Post	Pre	Post	Pre	Post	Pre	Post	Pre	Post	Pre	Post	Pre	Post
51.4483	52.241	12.3679	9.1257	18.1015	16.7613	18.1018	17.8281	21.6224	18.7128	4.64	3.7911	1.4053	1.2106	16.9691	18.5013	32.8163	33.8374
57.5041	60.8592	11.3722	11.9877	19.0355	17.474	19.8644	18.0848	19.7472	19.4539	3.8607	4.4535	1.3876	1.4761	19.4437	19.4614	34.5044	40.2017
63.4639	31.8835	10.3191	5.1199	17.526	7.0431	18.1608	7.2417	21.6718	7.0013	3.5351	1.7751	1.4295	0.6655	19.9604	8.0055	33.7389	15.0428
55.0638	56.9635	6.8639	8.4964	13.7736	14.7056	17.5809	17.2143	19.7363	20.4982	2.4564	2.7541	0.9169	1.2365	18.7701	19.408	34.6541	37.7891
57.5231	58.146	9.7598	9.8441	25.3821	18.1833	26.2432	16.4805	24.6719	14.093	3.1431	3.6201	1.1227	1.2314	25.4025	18.9564	52.2977	37.4357
55.5832	52.2752	6.7895	6.0524	17.4328	15.6364	21.4437	19.2359	24.5373	20.9733	2.5095	2.2803	1.131	1.0146	20.6408	19.8962	42.3291	32.1238
52.1908	49.9261	10.6222	9.0692	15.4174	19.1331	15.8104	20.2786	16.1666	21.1109	3.6744	2.8977	1.3	1.0475	16.0308	20.0288	30.0363	40.8526
60.8347	47.595	6.5154	5.374	17.6548	17.4961	22.7924	22.7523	22.5765	23.5678	2.9603	2.1434	1.0749	0.7862	22.0784	23.5156	31.3543	38.1328
50.9196	54.2742	6.7215	8.2277	21.0489	23.4995	23.7633	24.3491	22.8076	16.7613	2.5967	3.4173	1.1124	1.3334	24.8991	28.3159	37.3835	52.6407
40.8399	31.4033	5.3244	4.9997	14.7547	11.9459	16.3959	11.2073	14.7568	8.5376	2.2339	2.0611	0.7257	0.7473	15.4584	14.0104	23.3084	23.2292
56.6194	46.9548	12.9497	9.0119	20.6851	16.8027	20.4918	16.4998	19.1886	10.8991	4.7767	3.6075	1.4098	1.0233	19.4083	18.0864	34.551	32.4653
55.9758	45.2765	11.397	6.7115	21.9902	17.623	19.1331	15.6498	20.7822	11.7897	3.5589	2.5955	1.4717	0.8512	20.9591	17.4449	34.5491	32.5975
57.1462	38.8252	12.5608	3.6909	16.5226	12.2115	17.4778	12.6884	17.8326	8.0491	4.1055	1.7348	1.104	0.4864	17.7926	13.7595	33.6864	19.5448
49.8737	44.8453	7.3797	5.4942	17.3648	18.5952	19.5589	18.7377	20.0029	10.8735	2.8025	2.1064	1.0322	0.827	17.357	19.7265	27.705	31.2646
54.64189	47.96206	9.353079	7.371807	18.335	16.22219	19.77274	17.01774	20.43576	15.16582	3.346693	2.802707	1.187407	0.9955	19.65502	18.50834	34.49389	33.36843
5.470134	9.055208	2.647904	2.353454	3.097767	3.902275	2.951488	4.450438	2.882183	5.629804	0.809876	0.849829	0.220518	0.281712	2.997065	4.668246	6.746599	9.487812
